# Architectural Features and Resistance to Food-Grade Disinfectants in *Listeria monocytogenes*-*Pseudomonas* spp. Dual-Species Biofilms

**DOI:** 10.3389/fmicb.2022.917964

**Published:** 2022-06-09

**Authors:** Pedro Rodríguez-López, Juan José Rodríguez-Herrera, Marta López Cabo

**Affiliations:** Laboratory of Microbiology and Technology of Marine Products (MICROTEC), Instituto de Investigacións Mariñas (IIM-CSIC), Vigo, Spain

**Keywords:** antimicrobial resistance, benzalkonium chloride, biofilm, CLSM, disinfectants, electrolyzed water, *Listeria monocytogenes*, *Pseudomonas* spp.

## Abstract

*Listeria monocytogenes* is considered a foodborne pathogen of serious concern capable of forming multispecies biofilms with other bacterial species, such as *Pseudomonas* spp., adhered onto stainless steel (SS) surfaces. In an attempt to link the biofilms’ morphology and resistance to biocides, dual-species biofilms of *L. monocytogenes*, in co-culture with either *Pseudomonas aeruginosa*, *Pseudomonas fluorescens*, or *Pseudomonas putida*, were assayed to ascertain their morphological characteristics and resistance toward benzalkonium chloride (BAC) and neutral electrolyzed water (NEW). Epifluorescence microscopy analysis revealed that each dual-species biofilm was distributed differently over the SS surface and that these differences were attributable to the presence of *Pseudomonas* spp. Confocal laser scanning microscopy (CLSM) assays demonstrated that despite these differences in distribution, all biofilms had similar maximum thicknesses. Along with this, colocalization analyses showed a strong trend of *L. monocytogenes* to share location within the biofilm with all *Pseudomonas* assayed whilst the latter distributed throughout the surface independently of the presence of *L. monocytogenes*, a fact that was especially evident in those biofilms in which cell clusters were present. Finally, a modified Gompertz equation was used to fit biofilms’ BAC and NEW dose-response data. Outcomes demonstrated that *L. monocytogenes* was less susceptible to BAC when co-cultured with *P. aeruginosa* or *P. fluorescens*, whereas susceptibility to NEW was reduced in all three dual-species biofilms, which can be attributable to both the mechanism of action of the biocide and the architectural features of each biofilm. Therefore, the results herein provided can be used to optimize already existing and develop novel target-specific sanitation treatments based on the mechanism of action of the biocide and the biofilms’ species composition and structure.

## Introduction

*Listeria monocytogenes* is currently considered an issue of concern for public health (Quereda et al., 2021). This Gram-positive bacterial pathogen is the causative agent of human listeriosis, an uncommon disease causing high morbidity and mortality with symptoms that can vary from mild gastroenteritis and fever, to septicemia (Vázquez-Boland et al., 2001a,b). According to the latest One Health Report of the European Food Safety Authority (EFSA), the notification rate of listeriosis in the European Union was 0.42 per 100,000 population, representing a decrease of 7.1% in comparison with previous data (EFSA and CDC, 2021). Despite its low incidence, the mortality was 14.16% among confirmed cases and 20.48% among those patients that required hospitalization, especially among the so-called YOPIs (young, old, pregnant, and immunocompromised) group (EFSA and CDC, 2021).

The common route for the transmission of the pathogen into the population is via foodstuffs, especially meat/meat-based (Fabrizio and Cutter, 2005; Kramarenko et al., 2013) and fish/fish-based ready-to-eat (RTE) products (Ghorban Shiroodi et al., 2016; EFSA, 2018), and contamination occurs mainly at a processing level (EFSA and CDC, 2021). Environmentally, this pathogen can persist and survive by adhering to abiotic surfaces, such as stainless steel (SS), associated with other bacterial species forming multispecies biofilms (Elias and Banin, 2012; Willems et al., 2016; Møretrø and Langsrud, 2017). These are highly organized sessile structures of bacteria embedded in a self-produced polymeric extracellular matrix, which confers bacteria therein a higher resistance to environmental aggression such as desiccation, radiation, and antimicrobials, compared with its planktonic (i.e., free) counterparts (Li et al., 2021).

Among *L. monocytogenes*-accompanying species in biofilms, several authors have studied dual-species biofilms in co-culture with *Pseudomonas* spp. in terms of association capacity and resistance to biocides. Early studies in the field demonstrated that in *L. monocytogenes* Scott A–*Pseudomonas* sp. biofilms, the latter was able to predominate the structure, but that both were able to endure peracids, and the efficacy of such chemicals is negatively influenced by the presence of organic matter (Fatemi and Frank, 1999). Similarly, Saá Ibusquiza et al. (2012a) demonstrated that in 96-h-old biofilms grown on SS of *L. monocytogenes* CECT 4032 and CECT 5873 in co-culture with *Pseudomonas putida*, the resistance toward benzalkonium chloride (BAC) significantly increased for both species. Contrarily, Giaouris et al. (2013) reported that in *L. monocytogenes*–*P. putida* mixed-species biofilms, only the latter presented an increased resistance to continuous exposure to BAC, even though no apparent adaptation was present in either of the strains.

More recently reported data demonstrate that *L. monocytogenes* ATCC 19114 and ATCC 19115 are able to associate and grow at 10°C in polystyrene microplates with *Pseudomonas aeruginosa* and that this association allows both *L. monocytogenes* species to endure and survive against higher concentrations of ciprofloxacin and sodium hypochlorite (Yamakawa et al., 2018). In a similar way, Haddad et al. (2021) determined that the minimum biofilm eradication concentration (MBEC) toward BAC for *L. monocytogenes* increased from 6.2 ± 1.4 to 58.3 ± 7.5 μg ml^–1^ when co-cultured with *Pseudomonas fluorescens*, but that this MBEC did not significantly vary in co-culture with *Lactobacillus plantarum*, indicating that the first accompanying species provides *L. monocytogenes* some sort of protective effect that was not present with the latter.

Considering the complexity of biofilms’ structures, microscopy approaches have been used to study their architectural features. For example, epifluorescence microscopy coped with image analysis has been used for numerical spatio–temporal characterization of monospecies *L. monocytogenes* biofilms grown on SS (Mosquera-Fernández et al., 2014; Rodríguez-López et al., 2015). Other authors have used this approach to evaluate the cleaning and disinfection potential of pronase–BAC combined strategies against *L. monocytogenes*–*Escherichia coli* dual-species biofilms (Rodríguez-López et al., 2017b) and the development of tolerances to those treatments after continuous sublethal exposures (Rodríguez-López and Cabo, 2017).

In addition to the epifluorescence techniques, for in-depth biofilm studies requiring tridimensional characterization, confocal laser scanning microscopy (CLSM) approaches are preferred (Relucenti et al., 2021). As an example, Bridier et al. (2010) elegantly described the biofilm architecture of different strains of *E. coli*, *Enterococcus faecalis*, *L. monocytogenes*, *P. aeruginosa*, *Staphylococcus aureus*, and *Salmonella enterica*. In a similar way, Mosquera-Fernández et al. (2016) described the structural biofilm dynamics of three different *L. monocytogenes* strains analyzing the resulting CLSM micrographs with three different software tools. In addition, CLSM has been used to determine the biovolume of single- and dual-species biofilms of different food-borne pathogens, as well as their three-dimensional distribution with some species, such as *L. monocytogenes*, with a clear preference toward the bottom layers (Puga et al., 2014, 2018). The latter has been recently hypothesized to be one of the potential factors that could explain the higher resistance to biocides in *L. monocytogenes*-carrying biofilms.

Despite the abovementioned, the relationship between the structure of *L. monocytogenes* mixed biofilms and the resistance toward sanitizers still remains controversial. In this line, several authors have considered the biofilm complexity and its antimicrobial resistance as two related phenomena (Pan et al., 2006; Saá Ibusquiza et al., 2012a). On the contrary, Kocot et al. (2021) demonstrated that in *L. monocytogenes*–*E. coli* dual-species biofilms cultured in a batch (i.e., polystyrene plates) or fed-batch (i.e. CDC biofilm reactor) system, the resistance toward BAC was not determined by the structural characteristics of the biofilm itself but by the operational culture conditions in which the samples were grown.

Therefore, the generation of such knowledge would contribute to (i) giving valuable information to unravel the mechanisms of action of disinfectants on biological systems involving Gram-positive – Gram-negative mixed-species populations and the factors that influence the bactericidal potential of a given treatment and (ii) providing empirical data that could be used to optimize the amount of biocides deployed in a given sanitation treatment.

The main aim of the present work was to attempt to ascertain the link between the architectural features and the resistance toward two food-grade biocides in *L. monocytogenes*–*Pseudomonas* spp. dual-species biofilms grown on SS. Firstly, the cross-influence of *L. monocytogenes* and *Pseudomonas* spp. in mixed communities in terms of adhesion and final biofilm morphology was assessed via classical agar plating and epifluorescence microscopy, respectively. Next, a numerical characterization of three different *L. monocytogenes*-*Pseudomonas* spp. biofilms was carried out using CLSM coupled with image analysis. Finally, the susceptibility of the biofilms toward BAC and neutral electrolyzed water (NEW) was assessed by modeling the obtained dose-response experimental data.

## Materials and Methods

### Bacterial Strains

All strains used in this study are listed in Table 1. The strains of *L. monocytogenes* used in this study were isolated from three different origins: A1, E1, and G1, which were from food industry-related surfaces; L1, L7, and L34, which were isolated from fish products; and X1, X7, and X10, which were isolated from patients with human listeriosis.

**TABLE 1:** List of bacterial strains and codes used in this study.

Bacterial species	Strain code	Representative source	References
*Listeria monocytogenes*	A1	Environmental	Rodríguez-López et al., 2015
	E1	Environmental	Rodríguez-López et al., 2015
	G1	Environmental	Leite et al., 2006
	L1	Food	Rodríguez-López et al., 2019a
	L7	Food	Rodríguez-López et al., 2019a
	L34	Food	Rodríguez-López et al., 2019a
	X1	Clinical	Rodríguez-López et al., 2019a
	X7	Clinical	Rodríguez-López et al., 2019a
	X10	Clinical	Rodríguez-López et al., 2019a
*Pseudomonas fluorescens*	B52	Food	Allison et al., 1998
*Pseudomonas putida*	CECT 324	Environmental	Reference strain
*Pseudomonas aeruginosa*	CECT 110	Clinical	Reference strain

Regarding *Pseudomonas* spp., three different species were used as representatives of the same origins as for *L. monocytogenes*. Therefore, *P. putida* CECT 324, *P. fluorescens* B52, and *P. aeruginosa* CECT 110 were chosen as representatives for environmental (Wu et al., 2011; Meireles et al., 2013), food (Allison et al., 1998; Kumar et al., 2019), and clinical (Driscoll et al., 2007; Thi et al., 2020) sources, respectively.

Bacterial stock cultures were kept at −80°C in sterile brain-heart infusion broth (BHI; Biolife, Milan, Italy) containing 50% (*v v*^–1^) sterile glycerol. Similarly, working cultures were maintained at −20°C in sterile TSB (Cultimed, Barcelona, Spain) containing 50% (*v v*^–1^) sterile glycerol.

### Inocula Standardization

Before the cultivation of the biofilms, reactivation cultures were prepared by transferring 100 μl of each of the working cultures to a tube containing 5 ml of sterile TSB, incubated overnight at 37°C for *L. monocytogenes* and *P. aeruginosa* and 30°C for *P. fluorescens* and *P. putida*, and subcultured twice for the proper revivification of the cells.

Preinocula were prepared by adjusting the Abs_700_ to 0.100 ± 0.001 in sterile phosphate-buffered saline (PBS) using a 3000 series scanning spectrophotometer (Cecil instruments, Cambridge, United Kingdom). This absorbance corresponds to a cellular density of approximately 10^8^ CFU ml^–1^ according to previous calibrations.

### Biofilms Cultivation on Stainless Steel

Both *L. monocytogenes* monospecies and *L. monocytogenes-Pseudomonas* spp. dual-species biofilms were cultivated on 10 mm × 10 mm × 1 mm AISI 316 SS coupons (Comevisa, Vigo, Spain) following a protocol described by Rodríguez-López et al. (2017a), with minor modifications.

Preparation of the SS surfaces included washing with industrial soap to remove grease residues (Sutter Wash, Sutter Ibérica S.A., Madrid, Spain), thorough washing with tap water, final rinse with deionized water, and sterilization at 120°C for 20 min. Next, coupons were individually placed in a 24-well polystyrene flat-bottomed plate (Falcon, Corning, NY, United States).

In all cases, inocula were prepared by diluting the preinocula 1:10,000 in sterile TSB to obtain a final density of approximately 10^4^ CFU ml^–1^. For monospecies biofilms, 1 ml of the final suspension was used to inoculate each well containing a SS coupon. In the case of dual species biofilms, the corresponding inocula were 1:1 (*v v*^–1^) mixed, and 1 ml of the resulting mixture was used to inoculate the SS coupons. Next, plates containing coupons were placed at 25°C in static conditions for 2 h to allow initial adhesion and then in constant shaking at 100 rpm until needed.

Previous to any analysis performed, samples (SS coupons) were aseptically collected and immersed in 1 ml sterile PBS for 10 s to remove loosely attached cells.

### Epifluorescence Microscopy Assays

#### Influence of *Pseudomonas fluorescens* B52 in Dual-Species Biofilms

For this part of the study, all strains of *L. monocytogenes* (Table 1) were co-cultured with B52 strain and collected after 48 h, as described above. Then, the coupons were stained with FilmTracer™ LIVE/DEAD^®^ Biofilm Viability Kit (Life Technologies, Eugene, OR, United States) following manufacturer instructions to observe the final biofilm morphology and the distribution of live (green-emitting) cells and damaged/dead (red-emitting) cells. Biofilms were visualized under a Leica DM6000 epifluorescence microscope (Leica, Wetzlar, Germany) using a 40× dry objective and 10× ocular lenses. Representative images of each sample were acquired with a Leica DFC365 FX camera and the Metamorph MMAF software (Molecular Devices, Sunnyvale, CA, United States).

#### Morphology of *Listeria monocytogenes* L34 Biofilms in Co-culture With *Pseudomonas* spp.

In this second part of the microscopy assays, the variability of the resulting morphology of 48 h L34-*Pseudomonas* spp. dual-species biofilms were assessed. Therefore, three different dual-species biofilms, i.e., L34-CECT110, L34-CECT324, and L34-B52, were cultured and collected as described above. Samples were then stained by means of ViaGram™ Red+ Bacterial Gram Stain and Viability Kit (Life Technologies) following the manufacturer’s instructions. This stain allows in a mixed population the distinction of Gram-positive cells (red-emitting) with a counterstain with DAPI. Finally, samples were visualized under the epifluorescence microscope using a 63× water immersion objective, and the images were acquired as described in the previous section.

### Confocal Laser Scanning Microscopy Assay

To better determine the features of L34-*Pseudomonas* spp. biofilms, a CLSM approach was followed. For this, the same dual-species biofilms assayed in section “Morphology of *Listeria monocytogenes* L34 Biofilms in Co-culture With *Pseudomonas* spp.” were cultured and stained as described above. The biofilms’ CLSM images stacks were acquired in the Centre of Biological Engineering of the Universidade do Minho (Braga, Portugal) using the FluoroView application Software package (Olympus) and a FluoView FV1000 microscope (Olympus) equipped with a 60× oil immersion objective.

The resulting stacks were analyzed using COMSTAT 2.1 (Heydorn et al., 2000; Vorregaard, 2008) to obtain biomass (BM), maximum thickness (MxT), average thickness (AvT), and roughness coefficient (Ra), and with Confocal Uniovi ImageJ v1.51 software^1^ using the JaCoP plugin to obtain the Pearson’s correlation index (PCC) and Manders’ co-occurrence coefficients, M_1_ and M_2_, to obtain information regarding the distribution of both bacterial species within the biofilm (Aaron et al., 2018).

### Effect of Disinfectants on L34-*Pseudomonas* spp. Biofilms

The effects of two food-grade disinfectants, i.e. BAC (Guinama, Alboraya, Spain) and in-house made NEW, were tested on 48 h L34, L34 – CECT 110, L34 – CECT 324, and L34 – B52 samples.

In all cases, quantification of the remaining adhered viable cells (AVC) after the antimicrobial treatments was assessed by recovering them with two sterile cotton swabs pre-moistened in buffered peptone water (BPW; Cultimed, Barcelona, Spain). Swabs were placed in a tube containing 2 ml of sterile BPW and vigorously vortexed for 1 min to detach cells from the swab. The resulting suspension was serially diluted in sterile BPW and plated on tryptic soy agar (TSA; Cultimed, Barcelona, Spain). Results were expressed as the reduction in log CFU cm^–2^ compared to negative controls.

#### Benzalkonium Chloride

Benzalkonium chloride solutions were prepared in sterile deionized water at concentrations 50, 100, 150, and 200 μg ml^–1^ and kept at 4°C for no longer than 14 days. Therefore, 1 ml of each solution was applied at room temperature on 48-h samples (*n* = 3) for a contact time of 10 min. For negative controls, 1 ml of sterile deionized water was used instead. After BAC treatment, samples were neutralized by immersing them for 30 s in 1 ml of LPT neutralizing broth (composition per liter: 10 ml of a 34 g L^–1^ KH_2_PO_4_ buffer (pH 7.2); soybean lecithin: 3 g; Tween 80: 30 ml; Na_2_S_2_O_3_: 5 g; L-histidine: 1 g) and transferred to a fresh well. AVC quantification was performed as described above.

#### Neutral Electrolyzed Water

Neutral electrolyzed water was generated at room temperature using an Envirolyte EL-400 Unit model R-40 (Envirolyte Industries International Ltd., Estonia) according to the manufacturer’s instructions. Briefly, saturated NaCl solution and tap water were simultaneously pumped into the apparatus at an intensity of 20–25 A. Therefore, NEW was produced by appropriately mixing the anolyte solution [pH 2.0–3.0, oxidation–reduction potential (ORP) ≈ 1,200 mV] with the catholyte solution (pH 11.0–12.0, ORP ≈−900 mV) after electrolysis. ORP and pH of the resulting solutions were determined using a portable pH and REDOX 26 multimeter (Crison Instruments S.A., Barcelona, Spain). Total available chlorine (TAC) was determined by iodometric titration (APHA., 2012).

The resulting NEW had the following properties: TAC = 880 μg ml^–1^, pH 6.3, ORP = 940 mV. Next, TAC was adjusted with sterile deionized water to obtain working solutions of 100, 200, 400, 600, and 750 μg ml^–1^ and kept protected from light at 4°C for a maximum of 14 days.

For dose-response assays, 1 ml of each solution was applied at room temperature to 48-h samples (*n* = 3) for a contact time of 10 min, followed by neutralization and AVC quantification as described above. As in BAC assay for negative controls, 1 ml of sterile deionized water was used.

#### Data Fitting and Determination of Lethal Dose 50

Lethal dose 50 (LD_50_) can be defined as the dose of an antimicrobial required to achieve a killing of half of the initial bacterial population and was used as a parameter to determine the effect of disinfectants on dual-species biofilms. To assess this, fitting of the experimental values into the dose-response model was performed utilizing a modified Gompertz equation proposed by Murado et al. (2002) using the least-squares method (quasi-Newton) of the SOLVER function of Microsoft Excel 2016, as follows:


(1) $$R=K\,\left(e^{-e^{\left(b-\mathrm{cD}\right)}}-e^{-e^{b}}\right)$$

where *R*, biofilm reduction expressed in of log CFU cm^–2^; *D*, dose of disinfectant used; *K*, maximum logarithmic reduction (asymptote); *b*, parameter to be determined empirically (dimensionless); and *c*, specific inhibition coefficient (dimensions: inverse of the dose). Since Equation 1 modifies the resulting dose-response parameters by subtracting the intercept of the original equation, outcomes were further adjusted to obtain the new *K* value (*K*′), as follows:


(2) $$K^{\prime }=\lim_{D\to \infty }R=K\,\left(1-e^{-e^{b}}\right)$$

Finally, the LD_50_ value was calculated using Equation 3:


(3) $${\mathrm{LD}}_{50}=\frac{1}{c}\,\left\{b-\ln\left[\ln\frac{2}{1+e^{-c^{b}}}\right]\right\}$$

### Statistical Analysis

Two different tests were performed to assess significance among the obtained results at a confidence level of 95% (α = 0.05) or greater.

Specifically, to statistically compare the number of AVC of L34 in monoculture or co-culture with *Pseudomonas* spp., a paired two-tailed Student’s *t*-test was performed using Microsoft Excel 2016 statistical analysis tool.

Following this, to assess the significance of the CLSM parameters obtained, a one-way ANOVA with Bonferroni *post hoc* test was carried out using OriginPro 2021 v9.8.0.2000 (OriginLab corporation, Northampton, MA, United States).

Finally, in LD_50_ assays, the correlation coefficient (*r*) was obtained to determine the discrepancy between the logarithmic reductions’ experimental values and those expected with respect to the model.

## Results and Discussion

### *Pseudomonas fluorescens* B52 Influences the Final Biofilm Architecture Formed With *Listeria monocytogenes* Strains From Different Origin

**FIGURE 1:**
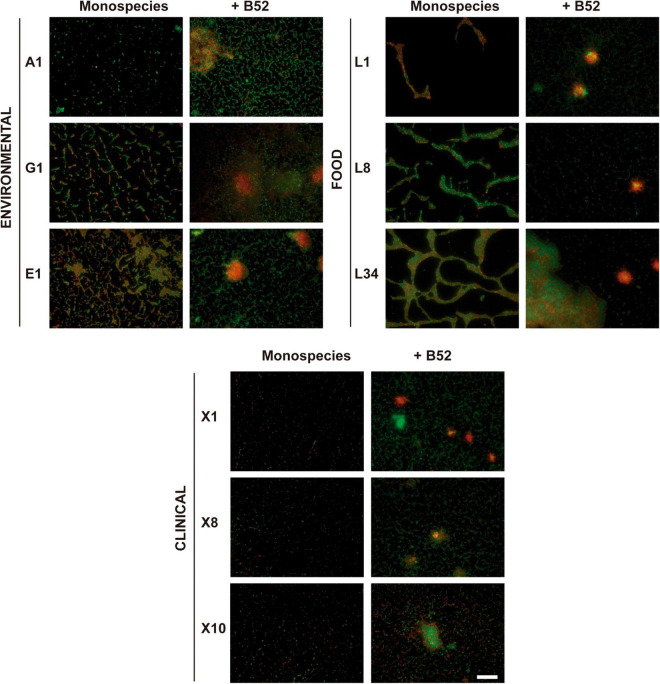
Influence of the accompanying strain. Representative 40×-field fluorescence micrographs after LIVE/DEAD staining of different *L. monocytogenes* 48-h-old biofilm cultured either in monospecies or dual-species with *P fluorescens* B52 on AISI 316 SS coupons. Scale bar, 50 μm.

*De visu* analysis of the epifluorescence microscopy images of monospecies *L. monocytogenes* 48 h biofilms grown on AISI 316 SS revealed a variety of growth on the coupon (Figure 1). Of note, all the *L. monocytogenes* strains used in the present study were chosen since a previous work demonstrated no statistically significant differences in the level of adhesion in terms of CFU cm^–2^ (Rodríguez-López et al., 2019a). Consequently, differences in the biofilm morphology, such as occupied area, would be attributed to factors other than the amount of viable-and-cultivable cells in the different biofilms.

In environmental strains (i.e., A1, E1, and G1), cells in the biofilm were distributed on the coupon forming a dense network predominated by live (green-emitting) cells according to the LIVE/DEAD^®^ staining, even though some red-emitting cells were present in the samples, implying that even though at early maturation stages, a certain number of cells can appear either damaged or dead (Figure 1). This form of sessile growth forming a sort of honeycomb-like structure has been previously described as the predominant way in which *L. monocytogenes* adheres to surfaces (Pilchová et al., 2014).

In a similar way, the biofilms formed by strains isolated from RTE fish products (i.e., L1, L8, and L34), were mainly characterized by a morphology in which the cells also formed a honeycomb-like structure but where the voids were visibly larger compared to the environmental strains (Figure 1). These voids were surrounded by dense groups of cells where live and damaged/dead cells were intermingled with a clear predominance of live cells in L8 and L34 strains, but not in L1 in which red-emitting cells were mostly present. Taking a closer look at the images of the biofilms formed by L8 and L34 strains, it can be observed that, even though similar, there is a trend in L34 to form clusters in which an accumulation of red-emitting cells is present (Figure 1). This observation is concomitant with the findings obtained in a study performed by Guilbaud et al. (2015) characterizing the biofilm morphological diversity of 96 *L. monocytogenes* strains. The authors demonstrated that not only *L. monocytogenes* mostly grows forming honeycomb-like shapes but clusters formed by a mixture of dead cells and matrix components are also common.

Lastly, biofilms formed by strains isolated from patients with human listeriosis (i.e., X1, X8, and X10) were characterized by groups of undamaged cells and, to a lesser extent, damaged/dead cells that were distributed throughout the surface that were not fully interconnected between them forming quasi-honeycomb structures. This fact was especially remarkable in X1 strain, where cell clustering was almost absent (Figure 1). This way of growth in which *L. monocytogenes* appears as stochastically sparse cells on a SS surface has been previously observed in other strains, such as *L. monocytogenes* CECT 4032 and CECT 5873 (Mosquera-Fernández et al., 2014).

The abovementioned structures were completely altered when all *L. monocytogenes* strains were co-cultured with *Pseudomonas fluorescens* B52 as displayed in Figure 1. The incorporation of the second strain deeply changed the resulting morphology of the biofilm turning it into a structure with an evident presence of big cell clusters surrounded by a network of mixed green- and red-emitting cells (Figure 1). These results are in line with those obtained previously with *L. monocytogenes*–*P. fluorescens* dual-species biofilms (Puga et al., 2014, 2016; Rodríguez-López et al., 2017a). It is well documented that *P. fluorescens* biofilms are characterized by the formation of microcolonies (Dynes et al., 2009; Wang et al., 2018). Thus, based on these results, it seems logical to think that *P. fluorescens* dominates the final structure in the dual-species biofilms, and *L. monocytogenes* would appear as a “passive” component of it. Of note, in mixed biofilms, there were a number of clusters present in the structure in which the local accumulation of damaged/dead cells was evident (Figure 1), being in line with previous observations (Rodríguez-López et al., 2017b). In this regard, Bayles (2007) described the role of dead/aged cells in biofilms, not only by acting as an anchoring strategy but also enhancing the stabilization of the final structure. Additionally, Jakubovics et al. (2013) demonstrated that dead and lysed cells can provide the biofilms with structural components, such as extracellular DNA (eDNA). This plays an essential role in *L. monocytogenes* biofilm formation as previously demonstrated (Harmsen et al., 2010a), pointing out that dead cells are not only a product of biofilm maturation/aging but also an integral part of the normal structure in *L. monocytogenes* dual-species communities.

### Influence of *Pseudomonas* spp. in the Final Morphology of *Listeria monocytogenes* L34 Dual-Species Biofilms: Adhesion and Two-Dimensional Analysis

**FIGURE 2:**
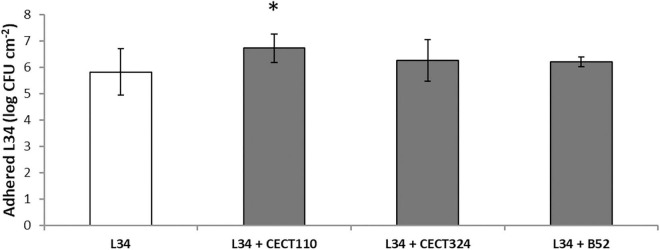
Plate count values of adhered viable cells (AVC) corresponding to 48 h control biofilms grown on AISI 316 SS coupons. Bars represent mean values (*n* = 15) expressed in CFU cm^– 2^ of *L. monocytogenes* L34 mono-species biofilms (void) and in dual-species biofilms with *Pseudomonas* spp. (gray). Error bars represent the samples’ standard error. Asterisk indicates statistical significance (two-tailed Student’s *t*-test, α = 0.05).

Firstly, the composition in terms of AVC of L34 biofilms alone and in combination with *Pseudomonas* spp. was compared (Figure 2). Despite being subtle, significant (*p* < 0.05) amount of AVC in L34 was observed when co-cultured with CECT110 when compared with the monoculture. In the rest of the samples, the level of adhesion of L34 did not present significant differences (Figure 2).

**FIGURE 3:**
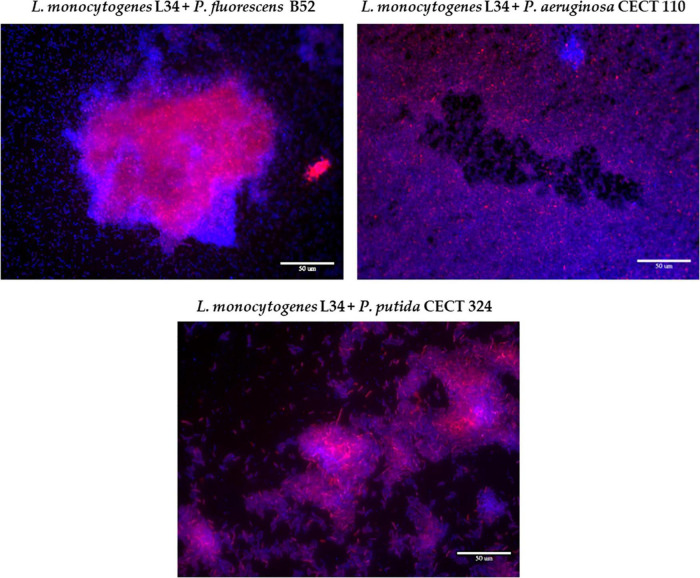
Representative epifluorescence 63×-field overlay images of the three dual-species biofilms assayed in this study, stained with ViaGram™ Red + Bacterial Gram Stain (ThermoFisher). Red signal: *Listeria monocytogenes* L34. Blue signal: *Pseudomonas* spp. Scale bar, 50 μm.

Epifluorescence images of the resulting biofilms were therefore analyzed to visualize the influence of a given *Pseudomonas* strain and compared it with the previous experiment (the reader is kindly referred to section “*Pseudomonas fluorescens* B52 Influences the Final Biofilm Architecture Formed With *Listeria monocytogenes* Strains From Different Origin” and Figure 1 for further details). As can be seen in Figure 3, the resulting morphologies of the dual-species biofilm were remarkably different depending on the accompanying *Pseudomonas* sp.

Co-culture with B52 gave rise to a structure with big cellular aggregates surrounded by sparsely distributed cells attached to the surfaces. Of note, these clusters presented a high red signal, meaning a local accumulation of L34 mixed with the accompanying species. This resulting morphology not only corroborated the results previously obtained with LIVE/DEAD staining (Figure 1) but also is in line with those previously obtained by Puga et al. (2014), observing that in *L. monocytogenes–P. fluorescens* biofilms, there is a tendency of the first to locally accumulate forming microcolonies with a high cellular density, whereas the rest of the biofilm is mainly occupied by the latter with cells randomly distributed all over the surface.

Similarly, a certain cellular clustering was present in L34-CECT324 samples, yet with some appreciable differences, as portrayed in Figure 3. In this particular case, cellular clumping was also evident although each cluster occupied a smaller surface compared to those in L34–B52 biofilms (Figure 3). This manner of growth has been previously observed by Hassan et al. (2004) in *L. monocytogenes*–*P. putida* dual-species biofilms on SS surfaces, concluding that in the absence of the latter, no cellular aggregation was present over the surface. Similarly, Saá Ibusquiza et al. (2012a) also observed this clusterization in dual-species biofilms of *L. monocytogenes* and *P. putida* grown on SS in comparison to polypropylene where, in the latter, much denser and compacted structures were formed.

Finally, the biofilm formed by L34-CECT110 was visually the one presenting the biggest differences compared to the two previous ones. This was characterized by a monolayer of cells in which both species were uniformly intermingled all over the surface (Figure 3). It has been previously reported that *P. aeruginosa* biofilms’ growth is mainly characterized by the formation of a dense and thick monolayer (Harmsen et al., 2010b; Rasamiravaka et al., 2015). Therefore, the fact that this structure remained unaltered despite the presence of L34 strain also strengthens the hypothesis that, in binary biofilms, *L. monocytogenes* does not influence the resulting structure, but adapts to the adhesion pattern of the accompanying species, as previously reported (Rodríguez-López et al., 2017a).

Following this first approach on the different structures of *L. monocytogenes*–*Pseudomonas* spp. biofilms, a CLSM analysis was performed to analyze their three-dimensional structures.

### Confocal Microscopy Analysis of *Listeria monocytogenes* L34 – *Pseudomonas* spp. Dual-Species Biofilms

In this second part of the study, the three-dimensional structural features of the three different *L. monocytogenes–Pseudomonas* spp. dual-species biofilms were assessed by CLSM coupled with image analysis.

**FIGURE 4:**
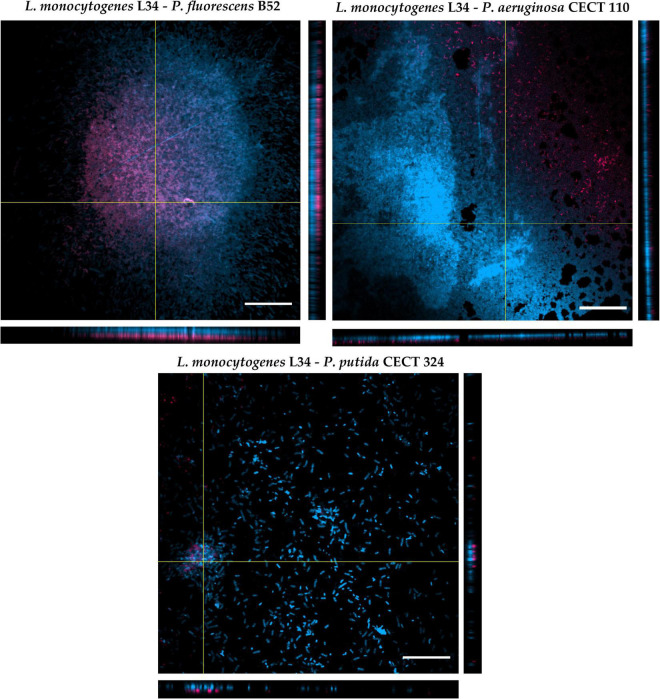
Orthogonal projections of CLSM of the three dual-species biofilms assayed in this study. The images were obtained using a 60× oil immersion objective. Red signal: *Listeria monocytogenes* L34. Blue signal: *Pseudomonas* spp. Scale bar, 20 μm.

As depicted in Figure 4, the CLSM zenital (*xy* plane) micrographs show how the different architectures of the biofilms obtained in the previous assay were repeated (see section “Influence of *Pseudomonas* spp. in the Final Morphology of *Listeria monocytogenes* L34 Dual-Species Biofilms: Adhesion and Two-Dimensional Analysis” and Figure 3), confirming once more the influence of *Pseudomonas* spp. in the final architecture of the biofilm itself and showing the abovementioned patterns of distribution. Additionally, orthogonal *xz* and *yz* planes clearly showed that L34, with respect to *Pseudomonas* spp., was located in the lower layers of the structure in all cases. Nevertheless, subtle differences in the way of the red and blue signals that were distributed along the images on the *z*-axis could still be appreciated. That is, whereas in co-culture with CECT110 and CECT324, the red signal corresponding to L34 strain did not exactly coincide with the blue signal corresponding to *Pseudomonas* spp., and appeared to be randomly intermingled, and in L34-B52 biofilms the red signal was almost coincident with the blue signal and located below it.

This phenomenon of cellular distribution, known as blanketing, initially described in *P. aeruginosa*–*Agrobacterium tumefaciens* dual-species biofilms (An et al., 2006), has been previously observed in a study conducted by Puga et al. (2014) with *L. monocytogenes* Scott A co-cultured with *P. fluorescens* ATCC 948. The authors demonstrated that, regardless of the temperature and the age of biofilms, *L. monocytogenes* appears located in the bottom layers of the biofilms. This way of growth is not only limited to *Pseudomonas* spp., but also in mixed culture with other Gram-negative species such as *E. coli* and/or *Salmonella* spp. (Almeida et al., 2010). Early studies dealing with *L. monocytogenes* mixed-species biofilms, attributed this fact to the already described slow growth of the pathogen with respect to other Gram-negative species, making *Listeria* become somehow covered or even masked by classical detection methods (such as selective agar plating) by the accompanying strain (Almeida et al., 2010; Langsrud et al., 2016). Nevertheless, a recent study demonstrated how planktonic cells of *L. monocytogenes* are able to invade and locate themselves in the bottom layers of an pre-formed *P. fluorescens* biofilm, taking advantage of the voids between the cellular aggregates left by the latter, and that this colonization was more successful at low temperatures (Puga et al., 2018). In addition to this, Maggio et al. (2021) showed that in dual biofilm with *P. fluorescens*, *L. monocytogenes* is not only able to better adhere to but over-produce extracellular matrix components compared with monospecies biofilms. Taken together, these data suggest that *L. monocytogenes* distribution within multispecies communities is not a random phenomenon, but appears to be actively regulated, also taking advantage of its facultative anaerobe and psychrotrophic characteristics, allowing the pathogen to associate with a wide diversity of microorganisms in a very structured manner (Rodríguez-López et al., 2015, 2019b).

Biofilms were further analyzed using COMSTAT 2.1. (Heydorn et al., 2000; Vorregaard, 2008) to get quantitative data on the mixed structures. The parameters obtained were BM (volume per area unit of field, representing an estimation of the amount of biofilm adhered to the coupon), AvT (mean of the heights in the biofilm), MxT (maximum value of thickness over the analyzed stacks), and Ra (variations of thickness throughout all the analyzed surface, giving information about the heterogeneity of the structure). These parameters have been described and used previously as good descriptors and can be easily interpreted in biological terms (Rodrigues and Elimelech, 2010).

Biomass values exhibited by the three dual-species biofilms did not present significant differences among them (Table 2). This indicates that, regardless of the *Pseudomonas* spp., the amount of biofilm attached to the surface of the SS coupon is similar. Thicknesses of the different structures revealed that despite all the structures that had similar MxT values, if AvT parameter is compared among structures, L34-CECT110 and L34-CECT324 biofilms presented differences between them but not with L34-B52 (Table 2). Of note, in those presenting significance, the differences of MxT vs. AvT are remarkable, especially relevant in L34-CECT324, indicating a high diversification in the levels of thickness throughout the colonized surface. This fact was further corroborated by the Ra coefficient (Table 2), which indicated that, in the experimental conditions assayed, L34-CECT324 may present a higher heterogeneity throughout the surface.

**TABLE 2:** Confocal laser scanning microscopy parameters of 48 h *L. monocytogenes*–*Pseudomonas* spp. mixed-species biofilms obtained after image analysis with COMSTAT 2.1. and colocalization analysis with JACoP (v2.0) plugin in ImageJ (see section “Epifluorescence Microscopy Assays” for further detail).

		L34 – CECT 110	L34 – CECT 324	L34 – B52
COMSTAT parameters	BM (μm^3^ μm^–2^)	0.61 ± 0.13 a	0.28 ± 0.06 a	0.57 ± 0.42 a
	MxT (μm)	4.96 ± 1.17 a	4.86 ± 1.01 a	3.15 ± 1.15 a
	AvT (μm)	1.32 ± 0.48 a	0.59 ± 0.24 b	0.69 ± 0.43 a,b
	Ra (dimensionless)	1.23 ± 0.07 a	1.67 ± 0.07 b	1.38 ± 0.39 a,b
Colocalization analysis	PCC	0.29 ± 0.14 a	0.55 ± 0.04 b	0.67 ± 0.14 b
	M_1_	0.06 ± 0.05 a	0.50 ± 0.07 b	0.56 ± 0.21 b
	M_2_	0.90 ± 0.05 a	0.70 ± 0.07 a	0.94 ± 0.04 a

*Each value represents the mean from each set of z-stack ± SD. In each row, different letters next to values mean statistically significant differences (one-way ANOVA; α = 0.05).*

*BM, biomass; MxT, maximum thickness; AvT, average thickness; Ra, roughness; PCC, Pearson’s correlation coefficient; M_1_ and M_2_, Manders’ co-occurrence coefficients.*

Despite the relevance of the microorganisms assayed, to the best of the authors’ knowledge, no other studies have been found in the literature that quantitatively characterize *L. monocytogenes*–*Pseudomonas* spp. biofilms grown on SS. Nevertheless, a number of articles regarding the study of the monospecies biofilms of the bacteria used in the present study have been published so far. In this line, the study published by Heydorn et al. (2000) described the formation of *P. putida*, *P. fluorescens*, and *P. aeruginosa* biofilms over time. Analysis of the structural patterns showed, and being in line with the results obtained in this study, that *P. aeruginosa* presented the highest values followed by *P. fluorescens* and *P. putida*. Moreover, it was demonstrated that Ra showed its highest value in the case of *P. putida*. The authors also described three different ways of growth characterized by a formation of cellular aggregates mixed with individual attached cells in *P. putida*, a higher coverage but less heterogeneous (lower Ra value) in *P. fluorescens* and a rapid and uniform colonization of the substratum in the case of *P. aeruginosa*, which is also observed in the microscopy images obtained in this study (Figures 3, 4).

Other studies demonstrated differences in COMSTAT values compared with those obtained in the present work. As an example, Ouyang et al. (2020) analyzed 24-h *P. putida* biofilms cultured in 96-well polystyrene plates yielding values of 12.04 ± 2.60 and 30.67 ± 10.01 μm for AvT and MxT, respectively, and 0.89 ± 0.073 for Ra. Similarly, Mah et al. (2003) obtained for 24 h *P. aeruginosa* biofilms BM values of 4.98 ± 3.18 μm^3^ μm^–2^, 5.08 ± 3.24, and 17.67 ± 2.47 μm for AvT and MxT, respectively, and 1.05 ± 0.31 for Ra. The fact that generally lower values of all the parameters analyzed obtained could be due to the orbital shaking acting in the detriment of the biofilm in two possible ways: (i) favoring the detachment of the cells because of shear stress and/or (ii) causing a rapid depletion of nutrients by *Pseudomonas* spp. as a consequence of the increased oxygen income in the culture medium which enhanced its overall growth (Kjelleberg and Molin, 2002). Apart from this, it is important to take into account that, even within strains, intrinsic structural variations occur making the overall comparison between samples to be very difficult (Heydorn et al., 2002).

**FIGURE 5:**
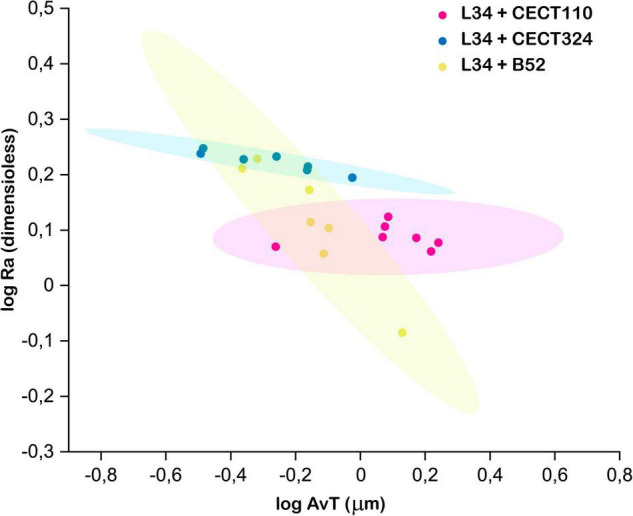
Clustering of the three structures using log AvT and log Ra values. Each spot of the scatter plot corresponds to one *z*-stack. Ellipses represent the 90% confidence for each data set. Two overlapping ellipses mean that a given pair of biofilms were not statistically different at the sampling time.

Next, AvT and Ra were plotted to assess the structural heterogeneity (Figure 5) since these parameters have been previously identified as good descriptors of biofilm structures (Heydorn et al., 2002). Before plotting, the values have been logarithmically transformed to stabilize their variances (Heydorn et al., 2002). Moreover, previous determination of covariance in each dual biofilm indicated that there is no correlation between them, and these two variables describe orthogonal features of the resulting structure (data not shown). As can be seen in Figure 5, L34–B52 confidence ellipse displayed overlapping with the ellipses of L34-CECT110 and L34-CECT324 that was not present in the latter two. This is indicative not that the biofilms can be considered equal between them, but that the resulting three-dimensional structures do not present statistically significant differences because of the uncertainty in the experimental conditions used.

To conclude the CLSM assays, a colocalization analysis was performed to determine whether the blue and red signals were interrelated among the image stacks. This was performed using Pearson’s correlation coefficient (PCC) defined as the degree of relationship between the fluorescent intensities in a particular image or region of interest (ROI) of an image (value between −1 and 1) (Wu et al., 2012) and Manders’ co-occurrence coefficients (M_1_ and M_2_), measuring the fraction of the pixels in a particular ROI or image that contains both blue and red signals (value between 0 and 1) (Barlow et al., 2010; Adler and Parmryd, 2012). All three structures showed positive PCC values suggesting that, in these mixed-biofilms, the relationship between both signals (i.e. bacterial species) is favored one over another (Table 2). This kind of behavior was somehow expected since it has been previously described that *Pseudomonas* spp. and *L. monocytogenes* are commonly found associated in food-related industrial premises sharing the same ecological niche (Møretrø and Langsrud, 2017; Fagerlund et al., 2021) eventually promoting a partnership based on cooperative phenomena and improving the overall fitness of the biofilm (Moons et al., 2009; Li et al., 2021). As a matter of example, it has been demonstrated that *L. monocytogenes* is able to stimulate *P. fluorescens* production of extracellular polymeric substances (EPS) causing an increase in the amount of biofilm matrix (Puga et al., 2018), which can be subsequently translated to a higher protection against desiccation and environmental aggression for both species (Elias and Banin, 2012). Of note, PCC value was significantly lower (*p* < 0.05) in L34-CECT110 compared with those obtained in the other two structures (Table 2), which can be attributable to the characteristics of the biofilm itself in which no cellular clustering is present.

After determining the mutualism in all biofilms assayed, co-occurrence coefficients were obtained to determine which fraction of the blue signal is actually contained in the red one (M_1_) and vice-versa (M_2_), giving information about the inter-dependence of each individual species (Barlow et al., 2010). As indicated in Table 2, and similarly to what occurred for PCC, M_1_ coefficient in L34-CECT110 was significantly lower compared to that obtained in the other two biofilms, indicating that in that particular structure, *P. aeruginosa* would be present in a particular location of the field regardless of the presence of *L. monocytogenes*. Conversely, the other two structures presented a higher M_1_ coefficient, suggesting a stronger relationship of CECT324 and B52 strains regarding L34 strain, which reinforces once more the aspects discussed above regarding the higher degree of interrelationship in those structures in which cellular clusters are present. Conversely, M_2_ coefficients (i.e., fraction of red contained in blue signal) did not present statistically significant differences between them, even though all presented remarkably high values (Table 2) indicative of a strong dependence of *L. monocytogenes* with regard to *Pseudomonas* spp. A feasible explanation for this is the fact that *Pseudomonas* spp. are well-known flagellar microorganisms and that these motility organelles are involved in the early stages of the adhesion processes (Elias and Banin, 2012; Palleroni, 2015). Hence, it could be hypothesized that in dual-species structures *Pseudomonas* spp. would adhere first to the coupon surface serving as a primary anchor for subsequent *L. monocytogenes* co-aggregation (Elias and Banin, 2012). In line with this observation, the study of Yamakawa et al. (2018) demonstrated how in *P. aeruginosa* ATCC 7700 in co-culture with *L. monocytogenes* ATCC 19114 and ATCC 19115, the final amount of measurable biomass in dual-species biofilms grown at 10°C depends primarily on the Gram-negative species. Similarly, Pang and Yuk (2019) demonstrated that despite lower numbers compared with monospecies biofilms, *L. monocytogenes* is able to adhere better to SS surfaces if these are pre-colonized with *P. fluorescens* ATCC 13525 with a decrease in the transfer rate of a 55.7% compared with monospecies biofilms.

It is important to remark that in industrial settings, the *L. monocytogenes*-carrying communities present tend to be much more complex in their composition (Fagerlund et al., 2017; Rodríguez-López et al., 2020) and that the final architecture, fitness and metabolic characteristics of the structure is a multifactorial process also depending on the environmental factors and nutrient availability, among others (Li et al., 2021). However, despite being simplistic, the observations made above could be considered as a starting point to establish empirical population dynamics models of multispecies since it quantitatively determines the interrelationships of both species after the analysis of the CLSM images, which highlights once more the usefulness culture-independent techniques, such as microscopy, for microbial ecology studies applied to food safety as previously reported (Almeida et al., 2011; Azeredo et al., 2016; Yuan et al., 2020).

Once the biofilms were numerically characterized, the biocidal effects of BAC and NEW were empirically determined in an attempt to establish a link between their final structure and their resistance toward them.

### Efficacy of Benzalkonium Chloride and Neutral Electrolyzed Water on *Listeria monocytogenes* L34 Cultured in Mono- or Dual-Species Biofilms

In this last part of the study, *K*′ value and the LD_50_ against disinfectants commonly used in food-related industrial premises (Fraser et al., 2021) were determined using a dose-response approach fitting the experimental data to a modified Gompertz equation (Murado et al., 2002).

In food-related premises, *L. monocytogenes*-carrying biofilms represent an issue of concern due to their inherent capacity to endure harsh environmental conditions while surviving common chemically based cleaning and disinfection protocols (Gray et al., 2021). To ensure proper sanitation standards on food and non-food surfaces of industrial premises, several chemical-based approaches have been used such as alcohols, peroxides, iodophors, or quaternary ammonium compounds (QACs) that have been routinely implemented in the sanitation standard operation procedures (SSOPs) (Wirtanen and Salo, 2003; Marriott and Gravani, 2006). Regarding the latter, BAC is one of the most known food-grade QACs used being a surface-active, stable agent, non-irritating, non-corrosive, and little affected by organic materials (Wales et al., 2021).

Besides QACs, chlorine-based chemicals, such as sodium hypochlorite (NaClO) or chlorine dioxide (ClO_2_), have been also used in postharvest industrial settings for sanitation of food and non-food surfaces (Aarnisalo et al., 2007). Their strong oxidizing nature is capable of rapidly disrupting the membrane components of bacteria causing unrepairable damage to the cell, and their relatively low cost of production and easiness of application, make them one of the most used disinfectants (Simpson and Mitch, 2021). Nevertheless, these chemicals have been an object of debate since their use has several drawbacks such as the limited action of the active forms, the release of chemical residues after treatment that can be combined with organic compounds forming toxic organochlorines, the generation of adverse effects to the organoleptic properties of food products and corrosion of industrial equipment, among others (Cap, 1996; Yan et al., 2021). Thus, NEW has been proposed as a cost-effective product for biofilm eradication (Vázquez-Sánchez et al., 2014; Rahman et al., 2016) since it has several advantages with respect to acidic or basic electrolyzed water, such as pH close to neutrality (6.5–7.5) and ORP values around 900 mV with the consequent lower metal corrosion risk of the equipment and more environmentally friendly than hypochlorite-based sanitizers (Arevalos-Sánchez et al., 2013).

**FIGURE 6:**
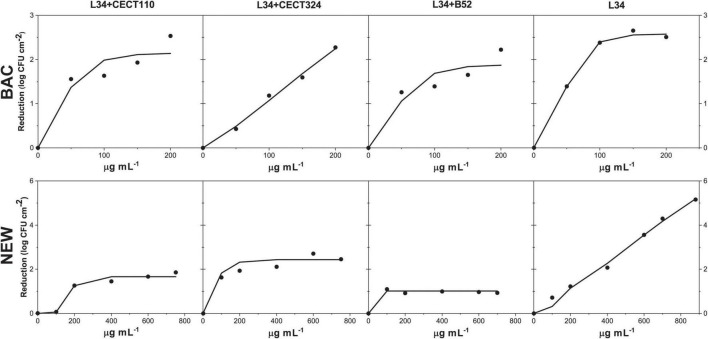
Fit of the dose-response experimental data expressed in log CFU cm^– 2^ reduction, in 48 h biofilms of L34 alone and co-cultured with CECT 110, CECT 324, and B52 after the application a solution of either BAC or NEW according to Equation 1.

Outcomes yielded in the present study gave a satisfactory fitting (*r* ≥ 0.95) in all samples processed (Figure 6 and Table 3). In general, *K*′ values indicated that BAC and NEW were most effective against monospecies biofilms than in dual-species samples. Of note, concentrations assayed did not permit to empirically determine the asymptote in L34 samples with NEW and L34-CECT324 with BAC, which would be the main cause of the high *K*′ values. However, the slope of the plot had a clear increasing trend with predicted values that can be considered valid based on the correlation coefficient obtained (*r* = 0.99). A feasible explanation underneath this higher effect in monospecies samples could be the simplicity of its three-dimensional morphology in which all the cells appear to be sparse throughout the surface of the coupon with all the surface of the cells exposed to the outer environment (Figure 1) in comparison to the dual-species biofilms in which cells, and although differently, are somehow compacted (Figures 3, 4), leaving less physical space susceptible to be attacked by NEW active forms. Supporting this, Cochran et al. (2000) demonstrated that there was a difference in the penetration of hydrogen peroxide (H_2_O_2_) between thick and thin biofilms in *P. aeruginosa*. Even though more resistant than the planktonic counterparts, in thin biofilms (cell density ≈ 3.5 log CFU cm^–2^) H_2_O_2_ penetration occurred directly, whereas in thicker biofilms, i.e., with more structure (cell density ≈ 7.6 log CFU cm^–2^), a barrier against the peroxide penetration was observed.

**TABLE 3:** L34 maximum logarithmic reduction (*K*′) and lethal dose 50 (LD_50_) expressed in log CFU cm^–2^ and μg ml^–1^, respectively, in 48 h biofilms of L34 alone and co-cultured with CECT110, CECT324, and B52 after the application of a solution of BAC or NEW.

	L34-CECT110			L34-CECT324			L34-B52			L34		
	*K*′	LD_50_	*r*	*K*′	LD_50_	*r*	*K*′	LD_50_	*r*	*K*′	LD_50_	*r*
BAC	2.14	37.85	0.95	4.92	193.84	0.99	1.88	44.34	0.95	2.57	47.35	0.99
NEW	1.66	187.02	0.95	2.43	55.76	0.97	1.01	2.29	0.99	7.84	724.04	0.99

*The fit of the values was carried out using Equations 1–3 (refer to section “Data Fitting and Determination of Lethal Dose 50” for further detail). In all cases, the correlation coefficient (r) is also presented.*

Comparing the biocidal effects in dual-species samples, L34 in co-culture with CECT324 was more susceptible to both BAC and NEW contact as demonstrated by the higher *K*′ values if compared with co-cultures with CECT110 and B52 (Table 3). Of note, in L34-CECT324 the susceptibility of L34 toward BAC was even higher than in monospecies samples displaying a reduction value 2.35 log CFU cm^–2^ higher (Table 3). These results are in contrast with the those of Saá Ibusquiza et al. (2012b) demonstrating that in 96-h *L. monocytogenes* CECT 4032–*P. putida* CECT 911 biofilms on SS, the first displayed a five-fold increase regarding the resistance to BAC if compared with their monospecies counterparts. However, in this case, the shorter growth times used can explain the lower resistance as it has been previously published that the matureness, i.e., age, of a *L. monocytogenes* mixed-species biofilm is one of the determining factors affecting its overall resistance (Rodríguez-López and Cabo, 2017). In another study, Giaouris et al. (2013) observed that in *L. monocytogenes-P. putida* dual-species biofilms, the latter displayed higher resistance to BAC compared with monospecies culture, even though there was no evidence of BAC-adaptations of neither of the species.

Among the two remaining biofilms, *K*′ values for both biocides were lower in L34-B52 when compared with L34-CECT110. It is known that the target of BAC is a cellular membrane (Aase et al., 2000) altering the alkyl chains therein, eventually provoking its breakage, leaking of the intracellular content, and subsequent death of the cell (Pereira and Tagkopoulos, 2019) and its mechanism of action is sequential, as follows: (i) adsorption onto the outer cell membrane (not applicable in Gram-positives), (ii) diffusion of BAC though the peptidoglycan wall, (iii) binding to the inner cell membrane and interaction with the phospholipids within, (iv) membrane disruption after the incorporation of the alkyl chain into the phospholipid bilayer, (v) leakage of the cytoplasmic content, and (vi) cellular death (Nagai et al., 2003; Alkhalifa et al., 2020). Considering this, the fact that L34–CECT110 and L34–B52 have very different architectures, but similar *K*′ values could be related to both the morphology of the biofilm itself in which L34, located in the bottom layers (Figure 4), would be somehow covered by *Pseudomonas* and therefore protected from BAC contact and because of the adsorption of BAC onto the *Pseudomonas* cell membranes. As Gram-negatives are generally more resistant to QACs (Mcdonnell and Russell, 1999), BAC could have remained sequestered onto the outer/inner membranes of the *Pseudomonas*, thus provoking a sharp decrease in the effective BAC concentration that could eventually reach L34 and undertake a biocidal effect. Supporting the latter, the study of García and Cabo (2018) modeled BAC effectivity in *E. coli* CECT 4622 demonstrating that the amount of biocide required to obtain a certain level of disinfection did not increase proportionately with the size of the inoculum, hypothesizing that the most plausible cause was the sequestering of the QAC onto the membranes, jeopardizing its action.

It is important to remark that, despite its low toxicity levels, the current European legislation in its Regulation (EC) 1119/2014 establishes a maximum level of residue of BAC of 0.1 mg kg^–1^ in certain products of plant and animal origin, which limits the application and the subsequent disinfection power (European Commission, 2014). Aside from this, several authors claim that the continuous misuse of these substances in industry, e.g., the application of QACs sub-lethal concentrations have provoked the generation of tolerant/resistant variants of certain microorganisms, e.g., *L. monocytogenes* and *Pseudomonas* spp., able to endure BAC treatment alone (Kanaris et al., 2021) or in combined treatments (Rodríguez-López and Cabo, 2017). For these reasons, modelistic dose-response approaches regarding the differences in the efficacy of BAC in different dual-species biofilms, such as the one herein presented, become a helpful tool to gather information to design more efficient sanitation protocols to be implemented in industrial hazard analysis and critical control points (HACCP) schemes.

Regarding NEW, the presence of *Pseudomonas* spp. on the top layers of the dual-species biofilms, could have provoked a decrease in the NEW active molecules available in the solution applied. Since active chlorine forms are rapidly inactivated by organic matter (Ayebah et al., 2006; Arevalos-Sánchez et al., 2013), the components of the matrix could have acted as protective agents inactivating them. Consistent with this is the fact that Pseudomonads are well-known to produce high amounts of protective alginate as a component of the biofilm matrix (Ciofu et al., 2022), and so this particular component could have conferred the biofilm an additional shield against the active components of NEW. In this line, Grobe et al. (2001) demonstrated that alginate-producing (mucoid) *P. aeruginosa* was about to endure higher concentrations of chlorinated water (>2 log CFU ml^–1^ survival) if compared to non-mucoid counterparts. Similarly, Xue and Seo (2013) showed that an alginate over-producing variant of *P. aeruginosa* PAO1 was able to produce more heterogeneous biofilm structures with a higher capacity to endure a chlorine disinfection treatment in a continuous flow system if compared with the non-mucoid and the wild-type variants.

All the above discussed can be further confirmed with the results obtained in COMSTAT 2.1. regarding the CLSM analysis (Table 2). Specifically, if Ra and BM values of the dual-biofilm are compared in relation to the minimum and maximum *K*′ values obtained in this study (Table 3), it is obtained on one side the L34-CECT110 biofilm where parameters suggested on average the highest biofilm out of the three (AvT = 1.32 ± 0.48 μm) and, on the other side, L34-CECT324 with an AvT = 0.59 ± 0.24 μm (Table 2). Additionally, although not significant, there was a difference in BM in which L34-CECT110 had the highest values and L34-CECT324 the lowest, indicative of a trend to have less dense structures in the latter case. These above mentioned differences are also indicative of the amount of exposed cellular membranes, which are higher in L34-CECT324 biofilms. Therefore, since the limitation of the biocide molecules diffusion through the matrix determines the effective amount of disinfectant reaching the lower strata and the subsequent biocidal effect (Bridier et al., 2011), in the latter case the lower density and the higher surface available to be attacked by BAC or NEW influenced the eventual effects as indicated by the *K*′ values (Table 3).

Finally, it is important to highlight that two of the major factors determining the efficacy of a given sanitation treatment are the dose of the agent and contact time. Therefore, if both disinfectants used are compared, and knowing that the contact time was constant in the experimental design, it can be observed that in NEW, *K*′ values were achieved much more rapidly compared to BAC and that the bactericidal effects did not improve if higher NEW concentrations were used (Figure 6). That is, in the contact time used if more NEW molecules were applied to the biofilm, it was not translated into a higher uptake. Therefore, the maximum reduction effects remained constant and equal to those achieved in the lower doses. Such differences are directly related to the mechanism of action of each disinfectant, fast in NEW but rapidly inactivated, and slow but sustained in time in BAC. Such phenomena should be taken into consideration, not only for the purposes of the present study, but also when designing novel sanitation regimes in a given industrial setting to determine dose, time, and other factors such as temperature to cost-effectively optimize it.

Although more research must be carried out involving different strains from different sectors, the present work provides a first step to unravel the link between biofilm structure and biocide resistance, which is of upmost importance to develop new and target-specific sanitation strategies.

## Conclusion

The outcomes of the present work further corroborates the observations previously made by our group, in which the final architecture of *L. monocytogenes*-carrying biofilms is deeply influenced by the accompanying species (Rodríguez-López et al., 2017a,b). Furthermore, it has been empirically demonstrated that *L. monocytogenes* L34 generally showed an increased resistance toward BAC and NEW when co-cultured with *Pseudomonas* spp. indicating that the latter conferred some level of protection. To conclude, it has been demonstrated that the effectivity of a given antimicrobial treatment depends not only on the bacterial composition but also on the mechanism of action the biocide considered. With this regard, NEW showed a highly reactive but unspecific and with a poor power of penetration in comparison to BAC, with a slower mode of action but more effective sustained along time as indicated by the empirical models.

On the whole, the results stress once more that for the correct design and optimization of a given antibiofilm treatment, it is of paramount importance to include factors other than the biocide itself. These include the composition and structure of the target biofilm, contact time, and temperature, among others.

## Data Availability Statement

The original contributions presented in the study are included in the article. Further inquiries can be directed to the corresponding author.

## Author Contributions

PR-L, JR-H, and MLC designed the research and carried out the data formal analysis and interpretation. PR-L lead the experimental work. PR-L and MLC wrote and revised the manuscript. All authors have read and agreed to the submitted version of the present article.

## Conflict of Interest

The authors declare that the research was conducted in the absence of any commercial or financial relationships that could be construed as a potential conflict of interest.

## Publisher’s Note

All claims expressed in this article are solely those of the authors and do not necessarily represent those of their affiliated organizations, or those of the publisher, the editors and the reviewers. Any product that may be evaluated in this article, or claim that may be made by its manufacturer, is not guaranteed or endorsed by the publisher.

## Footnotes

1
https://www10.uniovi.es/confocaluniovi/index.htm

## Abbreviations

AVC: adhered viable cells
AvT: average thickness
BAC: benzalkonium chloride
B52: *Pseudomonas fluorescens* B52
CECT110: *Pseudomonas aeruginosa* CECT 110
CECT324: *Pseudomonas putida* CECT 324
L34: *Listeria monocytogenes* L34
MxT: maximum thickness
M_1_: Manders’ co-occurrence coefficient 1
M_2_: Manders’ co-occurrence coefficient 2
NEW: neutral electrolyzed water
PCC: Pearson’s correlation coefficient
Ra: roughness coefficient
ROI: region of interest
SS: stainless steel
TAC: total available chlorine.
